# Noninvasive Quantification of *In Vitro* Osteoblastic Differentiation in 3D Engineered Tissue Constructs Using Spectral Ultrasound Imaging

**DOI:** 10.1371/journal.pone.0085749

**Published:** 2014-01-22

**Authors:** Madhu Sudhan Reddy Gudur, Rameshwar R. Rao, Alexis W. Peterson, David J. Caldwell, Jan P. Stegemann, Cheri X. Deng

**Affiliations:** Department of Biomedical Engineering, University of Michigan, Ann Arbor, Michigan, United States of America; University of Rochester, United States of America

## Abstract

Non-destructive monitoring of engineered tissues is needed for translation of these products from the lab to the clinic. In this study, non-invasive, high resolution spectral ultrasound imaging (SUSI) was used to monitor the differentiation of MC3T3 pre-osteoblasts seeded within collagen hydrogels. SUSI was used to measure the diameter, concentration and acoustic attenuation of scatterers within such constructs cultured in either control or osteogenic medium over 21 days. Conventional biochemical assays were used on parallel samples to determine DNA content and calcium deposition. Construct volume and morphology were accurately imaged using ultrasound. Cell diameter was estimated to be approximately 12.5–15.5 µm using SUSI, which corresponded well to measurements of fluorescently stained cells. The total number of cells per construct assessed by quantitation of DNA content decreased from 5.6±2.4×10^4^ at day 1 to 0.9±0.2×10^4^ at day 21. SUSI estimation of the equivalent number of acoustic scatters showed a similar decreasing trend, except at day 21 in the osteogenic samples, which showed a marked increase in both scatterer number and acoustic impedance, suggestive of mineral deposition by the differentiating MC3T3 cells. Estimation of calcium content by SUSI was 41.7±11.4 µg/ml, which agreed well with the biochemical measurement of 38.7±16.7 µg/ml. Color coded maps of parameter values were overlaid on B-mode images to show spatiotemporal changes in cell diameter and calcium deposition. This study demonstrates the use of non-destructive ultrasound imaging to provide quantitative information on the number and differentiated state of cells embedded within 3D engineered constructs, and therefore presents a valuable tool for longitudinal monitoring of engineered tissue development.

## Introduction

Bone tissue engineering approaches combine cells, biomaterials, and growth factors to recreate native bone tissue [1]. Traditionally, biochemical and histological assays are performed to monitor cell function and development in these engineered tissues. However, these techniques require sample processing and are destructive in nature, and therefore do not allow for an individual sample to be tracked as it develops. For example, traditional methods for characterizing cell number include manual counting chambers, automated cell counters, spectrophotometers, and flow cytometers [2], [3], [4]. These methods require a variety of sample processing steps including disruption of the tissue construct into constituents for counting (destructive in nature) and sample dilution. In addition, they may require specialized equipment and reagents that can be expensive. Importantly, most currently used measurement techniques describe only single timepoint, aggregate characteristics of the sample, and do not provide three dimensional (3D) spatial and temporal information.

Non-destructive approaches based on confocal microscopic imaging to count cell nuclei have been used to provide 3D assessment of cell numbers [5]. However, such techniques require high quality microscopy images, are time consuming, and involve complex processing algorithms to acquire entire spatially registered 3D images of the construct. Magnetic resonance imaging (MRI) and micro-computed tomography (µ-CT) techniques have been used to estimate bone mineral densities [6], [7]. However, these methods require the use of calibration phantoms and involve long data acquisition times [8]. Long exposures to X-ray may affect cell-seeded constructs in terms of the structure, viability, and cellular development of the constructs. Conventional MRI imaging systems do not provide the ability to study the microstructural details of 3D engineered tissue constructs due to their low resolution. Therefore there is a need for non-destructive imaging and characterization modalities, capable of providing both spatial and temporal information of engineered tissues as they develop *in vitro*. Such methods would greatly facilitate the translation of tissue engineering products from the lab to the clinic.

Ultrasound imaging is a widely used non-invasive and non-destructive method that has the potential for quantitative evaluation of tissue development both *in vitro* and *in vivo*. It has been reported recently that ultrasound can be used to quantify cell number in BMSC/β-TCP composites using a grayscale equivalent parameter [9]. Fite et al. used an ultrasound method to monitor the chondrogenic differentiation of equine adipose stem cells in 3D poly(lactide-co-glycolide) scaffolds [10] by correlating signal attenuation measured through gray scale image analysis to extracellular matrix (ECM) deposition, which was considered to be a marker of cell differentiation. Kreitz et al. tracked collagen deposition by myofibroblasts in fibrin tissue constructs over an 18 day culture period [11]. Their quantitative analysis correlated observed gray scale values to ECM deposition as measured by hydroxyproline content. Ultrasound has also been used as a tool to measure the mechanical properties of agarose hydrogels as they develop over time [12], by correlating material properties such as elastic modulus with obtained acoustic properties.

Ultrasound propagation and acoustic scattering in a tissue volume depend on tissue microstructure, composition, and physical properties such as density and compressibility. Therefore, backscattered ultrasound signals may be used to extract information about the structure and composition of the tissue under investigation, as well as its mechanical and physical properties. Although tissue properties such as speed of sound, acoustic attenuation and the tissue volume can be calculated directly from the backscattered radiofrequency (RF) data, tissue microstructural details are not apparent from the raw RF signals. Tunis *et al.* [13] studied the envelope statistics of ultrasound backscatter signals from cisplatin-treated aggregated acute myeloid leukemia (AML) cells and evaluated the applicability of various statistical distribution functions to model the envelope histograms. They reported that shape parameters of the generalized gamma distribution function were sensitive to structural changes within cells induced by the drug.

Quantitative ultrasound imaging methods using spectral analysis of the RF signals have been developed to extract additional parameters for enhanced tissue characterization. The power spectrum of the backscattered RF data includes information about tissue microstructure, and the spectral regression parameters can be related to scatterer properties such as effective sizes, concentrations and acoustic impedances [14], [15]. Spectral slope has been shown to depend on the scatterer size, whereas mid-band fit (MBF) relates to the size, concentration and relative acoustic impedances of the scattering elements [14]. Spectral analysis has been used in various applications, including characterization of plaque composition by intravascular ultrasound (IVUS) [16], [17], lesions induced by high intensity focused ultrasound (HIFU) [18], [19] and RF ablation [20]. Spectral parameters have also shown the ability to identify changes in tissue state for prostate, breast, pancreas, lymph node, and other cancer types [21], [22], [23], [24], [25], [26], [27]. Oelze *et al*. [22] developed methods to differentiate and characterize rat mammary fibroadenomas and 4T1 mouse carcinomas by estimating scatterer properties from backscatter RF signals in the spectral domain with a Gaussian form factor model [28].

The use of high frequency (20–60 MHz) ultrasound imaging has provided higher spatial resolution than conventional ultrasound imaging (5–15 MHz) in diagnostic radiology [23]. Kolios *et al.* have developed spectral analysis technique to characterize the properties of cell aggregates that were used as simplified models of tumors [23], [29], to detect cellular changes with high spatial resolution and sensitivity after exposure to chemotherapy drug treatments [30]. They found that ultrasound backscatter intensity and spectral slope increased due to treatment, which was interpreted as a consequence of the decrease in effective scatterer size of cell aggregates. The use of higher ultrasound frequency imaging, with corresponding ultrasound wavelengths in the order of 100 µm, permits sensing of changes in cell nuclei and cell structure.

In order to achieve non-invasive and quantitative assessment of engineered tissue constructs with high spatial resolution, we have implemented a high frequency spectral ultrasound imaging (SUSI) technique, and have validated its use to characterize the composition and structure engineered tissue constructs. In previous work, we used the spectral MBF and slope parameters to measure the quantity and spatial distribution of particulate hydroxyapatite in acellular collagen hydrogels [31]. We observed a strong correlation between MBF and mineral concentration, and between the spectral slope and particle size. The amount of mineral deposited from simulated body fluid on acellular collagen constructs over a period of 3 weeks was also studied using spectral parameters, and showed strong correlation with MBF.

The work presented here extends our previous study using high resolution SUSI to quantitatively characterize the osteogenic differentiation of MC3T3 mouse pre-osteoblast cells seeded within 3D collagen-based engineered tissues. MC3T3 cells are a well characterized mineralizing cell type that can be induced towards the osteogenic lineage with the addition of a defined set of supplements in the culture medium [32]. Collagen is a widely used biomaterial in orthopaedic tissue engineering due to its ability to support cell attachment and proliferation, as well as to serve as an osteoconductive and osteoinductive matrix [33]. In this study we non-destructively quantified the bulk properties of the hydrogels, including speed of sound, acoustic attenuation and volume compaction, and tracked these parameters over time. Microstructural properties of the cell-seeded constructs, including cell size, cell number, and cell differentiation, were also assessed using spectral ultrasound and were compared to data generated by traditional biochemical assays and confocal fluorescence imaging. This study demonstrates that SUSI can be used to non-destructively characterize cell-seeded engineered tissue constructs longitudinally over time with high spatial resolution.

## Materials and Methods

### Cell Culture

Mouse pre-osteoblast MC3T3-E1 (generously provided by Dr. R.T. Franchesci, University of Michigan) were cultured in α-MEM without ascorbic acid (Life Technologies, Grand Island, NY) supplemented with 10% fetal bovine serum (FBS; Life Technologies) and 1% penicillin and streptomycin (PS: Life Technologies) and used at passage 8. Media was changed every other day.

### Collagen Hydrogel Synthesis

Three-dimensional (3D) collagen hydrogels were created as previously described [31]. Briefly, collagen type I (MP Biomedicals, Solon, OH) was prepared at 4.0 mg/ml in 0.02 N acetic acid. Collagen hydrogels (2.0 mg/ml final concentration) were formed by mixing 10% Dulbecco's modified Eagle's medium (DMEM; Life Technologies), 10% FBS, 20% 5X-concentrated DMEM (stock concentration), 10% 0.1 N NaOH (Sigma Aldrich, St. Louis, MO), and 50% collagen stock solution. 500 µL of the mixture was then pipetted into a 24-well plate and allowed to gel for 30 mins at 37°C. Cells were encapsulated within the hydrogels at the time of gelation at a concentration of 1.0×10^6^ cells/ml.

After gelation, hydrogels were moved into a 6-well culture plate containing α-MEM supplemented with 10% FBS and 1% PS to allow cells to compact their matrices. After 24 hours, the media was changed with either a control media or osteogenic media containing 10 mM beta-glycero phosphate (β-GP; Sigma) and 50 µg/ml ascorbic acid 2-phosphate (Sigma).

### Cell Viability

Cell viability was visualized and quantified as previously described [34]. At days 1 and 21, cell-seeded hydrogels were washed 3X in phosphate buffered saline (PBS; Life Technologies) for 5 mins and then incubated in 4 µM calcein-AM (EMD Millipore, Billerica, MA) and 4 µM ethidium homodimer-1 (Sigma) in PBS for 45 mins. Constructs were washed 3X in PBS prior to imaging on a Nikon A1 Confocal Microscope (Nikon Instruments, Melville, NY). Cell viability was quantified using ImageJ software (National Institute of Health, Bethesda, MD).

### Fluorescence Staining

At days 1 and 21, cells were stained for their actin cytoskeleton and nuclei. Hydrogels were washed 2X in PBS for 5 mins/wash and then fixed in zinc-buffered formalin (Z-Fix; Battle Creek, MI) for 10 mins at 4°C. Gels were washed another 2X in PBS and then permeabilized using 0.5% Triton-X 100 (Sigma) in PBS for 20 mins at room temperature. Constructs were washed again 2X, and then incubated in a solution containing 165 nM AlexaFluor 488 phalloidin (Life Technologies) and 10 nM fluorescent DAPI (Life Technologies) in 1% bovine serum albumin (BSA; Sigma) in PBS for 45 mins. Hydrogels were washed again prior to imaging.

### Biochemical Assays

Cellular DNA content and calcium were quantified as previously described [35]. For DNA quantification, hydrogels were collected and degraded overnight in 10 mM Tris-HCl (Sigma) containing 0.6 mg/mL collagenase type I (MP Biomedicals), 0.2% IGEPAL (Sigma), and 2 mM phenylmethanesulfonylfluoride (Sigma). DNA was then measured using the PicoGreen DNA assay (Life Technologies). Calcium secretion was assayed by first dissolving the constructs in 1 N acetic acid (Sigma) overnight. The cell-hydrogel lysate was then assayed using the ortho-cresolphthalein (OCPC) method [31].

### Phantom Studies

Agar phantoms embedded with Polybead® microspheres (Polysciences, Inc., Warrington, PA) of different diameters at various concentrations were used to validate the size and concentration estimation from SUSI. Polybeads were added to the 2% agar solution at 45°C and thoroughly mixed to disperse them uniformly throughout the phantom. Four polybead diameters of interest were chosen: 6, 10, 16 and 25 µm. For each polybead size, phantoms with four different concentrations of polybeads were made. Each phantom was approximately 2000 mm^3^ in size.

### Ultrasound Imaging and Backscattered Signal Acquisition

**Figure 1:**
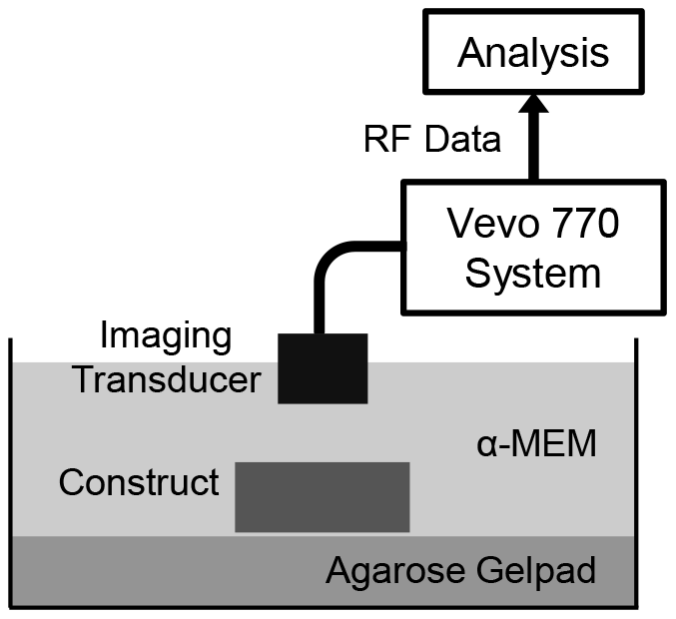
Schematic of experimental setup used for spectral ultrasound imaging (SUSI) of engineered tissue constructs.

Figure 1 shows a schematic diagram of the ultrasound imaging setup. A gel slab with 8% agarose (Sigma) was placed at the bottom of a 60 mm Petri dish to reduce ultrasound reflection from the bottom of the dish. The dish was then filled with α-MEM at room temperature and the constructs were placed on top of the agarose gel pad. Ultrasound imaging was performed using a Vevo 770 (VisualSonics Inc., Toronto, Canada) and an RMV 708 imaging probe with a nominal 55 MHz center frequency, 20–75 MHz bandwidth (−6 dB), 4.5 mm focal distance, and 1.5 mm depth of focus (−6 dB). Ultrasound B-mode imaging was performed with the ultrasound beam focus placed 0.5 mm below the top surface of each sample. The interval between adjacent A-lines in the B-scans was set at 31 µm. 3D imaging of the construct was performed by acquiring a series of B-scans with 200 µm interval between adjacent scans across the tissue construct using a computer-controlled automatic translational stage. The 3D image data were used to estimate the volume of the construct. The backscattered radiofrequency (RF) signals of all ultrasound images were acquired at a sampling rate of 420 million samples/s. For estimating the speed of sound and acoustic attenuation of the construct, backscattered RF data with ultrasound focus placed at the gel pad surface were collected with and without the presence of a construct.

### Ultrasound Imaging Analysis

#### 1) Construct Volume

A semi-automated segmentation procedure and edge detection algorithm from the Vevo 770 system were used to detect the contour of the construct in a B-image. The volume of the construct was then calculated as the volume within the contours defined from each of B-mode images separated by 200 µm in 3D image data.

#### 2) Speed of Sound in Construct

A grayscale parameter [31] was computed from the RF data of a B-scan. The time of travel of the ultrasound pulse from the imaging transducer to the construct top surface (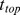), bottom surface (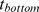), and the agar gel pad surface (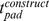) was determined based on grayscale thresholding using an automated algorithm. The time of travel to the agar gel pad without the construct (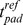) was also determined and used as the reference. Assuming the speed of sound in the surrounding fluid medium (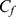) to be 1480 m/s, the thickness of the construct (*L*) was determined as
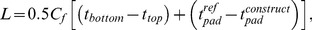
(1)and speed of sound in the tissue construct 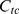 as
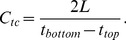
(2)


#### 3) Attenuation

Frequency dependent attenuation in dB/cm was calculated as:
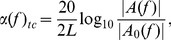
(3)where |*A(f)|* and *|A*_0_ *(f)|* are the spectral magnitudes of the RF signal from the gel pad surface with and without (reference) the presence of construct respectively. The slope of *α* against *f* was estimated by a linear fit between 20–55 MHz to yield the attenuation coefficient in the construct in dB/(cm-MHz).

### SUSI analysis

#### 1) Scatterer Size

The calibrated power spectrum of the RF signals for each A-line was obtained using linear regression to find the spectral parameters, i.e., the slope (*m*') and the mid-band fit (*MBF*') within a − 9 dB bandwidth [31]. The spectral parameters were corrected for the attenuation (*α,* dB/(cm-MHz)) of the tissue construct as *m = m*'*+2αz* and *MBF = MBF*'*+2αzf_c_* where *z* is the ultrasound propagation distance in the tissue construct. Ultrasonic spectral parameters have been related to the system factors and the physical properties of effective acoustic scatterers in tissue [14]. As described previously [14], spectral slope represents a parameter associated with scatterer radius (*a*), its geometry (*n*) and the center frequency of the imaging transducer (*f_c_*) and bandwidth (*b*), and is given by:
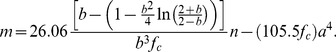
(4)


Thus, the scatterer radius can be calculated as:
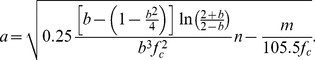
(5)


#### 2) Acoustic Concentration

The spectral MBF depends on an additional parameter, the acoustic concentration (*CQ^2^*),
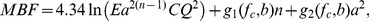
(6a)

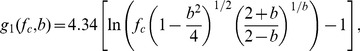
(6b)

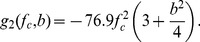
(6c)where *C* is the number concentration of the acoustic scatterers (/mm^3^), *Q* is the relative acoustic impedance, and *E* is a shape-dependent parameter. Eq. (6) can be rearranged to obtain *C* as
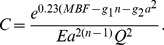
(7)


The relative acoustic impedance, *Q*, of MC3T3 cells was estimated from a known number concentration, *C*, of cells in constructs.

#### 3) Deposited calcium by cells

Differentiation of MC3T3 cells was assessed by detecting and quantifying the mass of the calcium deposited [36]. The mass of calcium at day 21 was calculated by comparing the relative acoustic impedance of the scatterers at day 0 to day 21. The relative acoustic impedance is defined as 
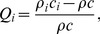
(8)where *ρ* and *ρ_i_* are the mass densities of the ECM and scatterers on the *i* ^th^ day, *c* and *c_i_* are the speed of sound in the ECM and scatterers on *i* ^th^ day respectively. On day 21, the presence of deposited calcium around the cells will increase relative acoustic impedance of the scatterer. With known relative acoustic impedance of the scatterer on day 0 (cell alone without calcium) and day *i* (cell and calcium), the mass of secreted calcium can be calculated as (derivation in Appendix S1.)
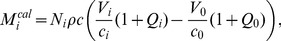
(9)where *N_i_* is the total number of cells on day *i* and *V_i_* is the volume of the net scatterer on day *i* and is approximately 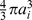.

#### 4) SUSI Parametric Images

The spatial distribution of scatterer features (scatter size and calcium concentration) within a construct was represented as parametric images where each pixel within a B-mode image was marked with a color that corresponded to the values of the scatterer size or calcium concentration.

### Statistical Analysis

Analysis of the scatterer size and concentration was carried out in an element volume with dimensions of 0.6 mm×5.0 mm× thickness (∼1 mm) throughout the construct. Results are presented as mean ± standard deviation. Normalization test was carried out successfully with *p*<0.01 on the data wherever statistical analysis was made. Statistical comparisons between any two parameters were performed using Student's t-test for paired samples and the differences were considered significant at a level of *p*<0.05.

## Results

### Validation of SUSI Estimation of Scatterer Size and Concentration

Verification of SUSI technique was performed using agarose phantoms embedded with polystyrene microspheres of known size and concentration. As shown in Table S1, experiments were performed on phantoms with 4 different concentrations of Polybead® polystyrene microsphere (Polysciences Inc.) of different sizes (6, 10, 16 and 25 µm diameter). The SUSI method was able to detect the scatterer (polystyrene spheres) size and concentration in these phantoms, validating the SUSI estimation protocol. Additional validation of SUSI for estimation of cell size was performed using MC3T3-seeded engineered tissue constructs on day 0 with four known concentrations of cells (Supplemental Figure S1). Constructs with a known concentration of cells (2×10^6^ cells/ml) were used to estimate the relative acoustic impedance of the MC3T3 cells via SUSI analysis, providing a value of approximately 0.6. SUSI was also used to estimate cell size, and the estimated cell diameter was approximately 14 µm (*n* = 9, range  =  ≈13.5–15.5 µm). The estimated diameter decreased slightly with increasing cell concentration (Figure S1E), possibly due to increased compaction of the constructs at higher cell concentration. The estimated relative acoustic impedance was then used to estimate the cell concentration of other constructs with prepared at different cell concentration (0.5, 1 and 5×10^6^ cells/ml) at day 0. These data are shown in Figures S1E, F, and show a good linear fit between the estimated concentration and the true concentration (R^2^ = 0.92). These data confirmed the ability of SUSI to detect particle size and concentration in hydrogel constructs.

### Virtual Histology for Longitudinal Monitoring of Tissue Constructs

**Figure 2:**
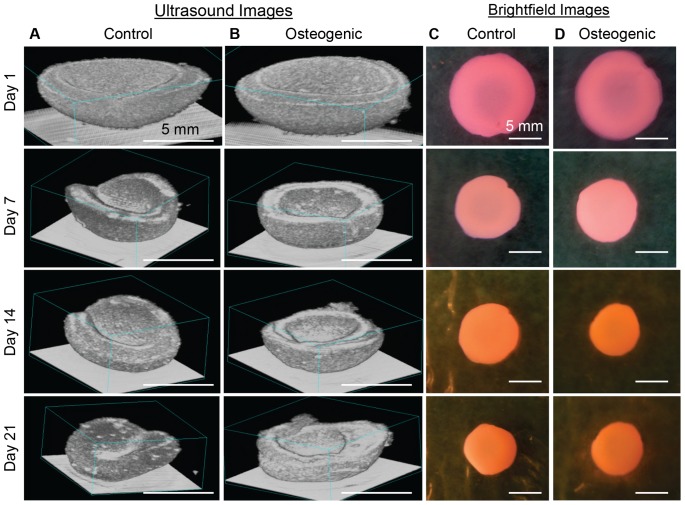
Longitudinal monitoring of MC3T3 cells seeded in collagen constructs. 3D rendered ultrasound backscattered images of the constructs in (A) control and (B) osteogenic media on day 1, 7, 14 and 21 of the development process. Brightfield images of corresponding constructs are shown in (C) and (D).

Imaging using the Vevo 770 system at 55 MHz achieved rapid and non-invasive 3D ultrasound imaging of MC3T3-seeded collagen constructs. During ultrasound imaging, the constructs in α–MEM media were removed from the incubator for less than 20 minutes. Figure 2 shows an example of non-destructive longitudinal monitoring of the progression and development of cell seeded collagen constructs incubated in control and osteogenic media. The 3D rendered ultrasound images, constructed on Visualsonics Vevo 770 imaging system, at different time-points (day 1, 7, 14 and 21) clearly show a reduction in the volume of the constructs over time (“gel compaction”) in both control and osteogenic media. Constructs adopted a symmetrical concave shape from day 1, which is a typical result of cell-mediated gel compaction. In addition, constructs in osteogenic medium became more echogenic than those in control medium, indicating changes occurring during development in osteogenic medium.

**Figure 3:**
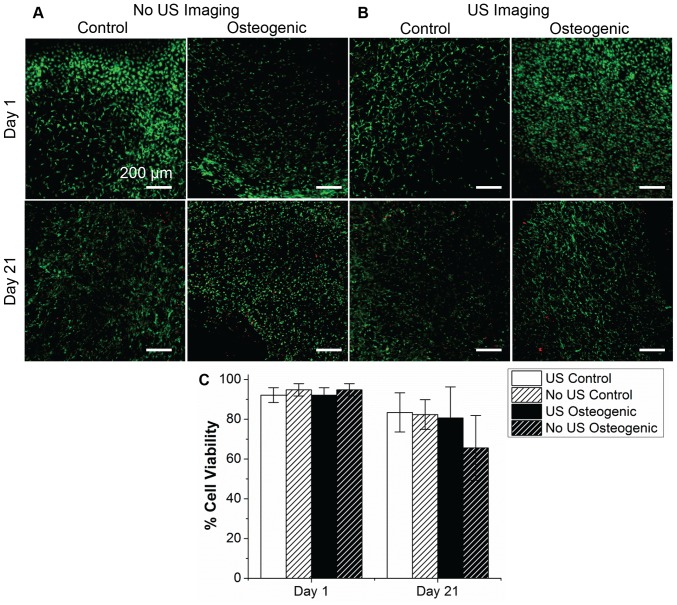
Comparison of cell viability at day 1 and 21 of MC3T3 cells seeded in collagen constructs, in either control or osteogenic media (cytoplasm of living cells is stained green, nuclei of dead cells is stained red). Constructs in (A) were not imaged using ultrasound, while those in (B) were imaged using ultrasound. Bar plot in (C) shows quantification of cell viability calculated from the images. Scale bar  = 200 µm. Best viewed in color.

Cell viability in the constructs with and without ultrasound imaging was compared to assess possible effects of ultrasound imaging. As shown in Figure 3, viability was greater than 90% at day 1 in all of the samples and greater than 70% in all samples at day 21. There were no statistical differences between the samples with and without ultrasound imaging at either time point, indicating that exposure to ultrasound imaging did not affect cell viability.

### Measurement of Construct Volume, Speed of Sound, and Acoustic Attenuation

**Figure 4:**
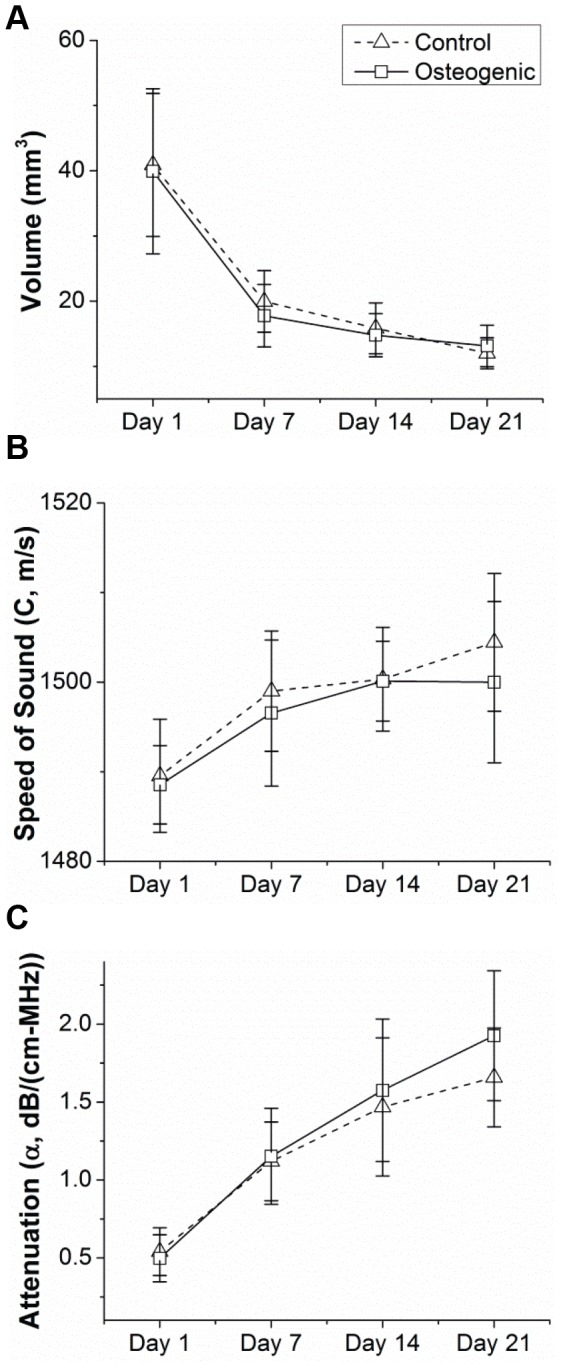
Backscatter analysis of MC3T3-seeded constructs in control and osteogenic media over time in culture. Quantification of (A) construct volume, (B) speed of sound, and (C) attenuation at each time-point.

As shown in Figure 4, a significant decrease in construct volume to about 25–30% of the original volume occurred between days 1 and 7, and the construct volume then stabilized between days 7 and 21. No statistically significant differences in construct volumes were detected between control and osteogenic media at any of the time-points. There was a slight increase and then plateau in the speed of sound over development time for constructs in both control and osteogenic media. The acoustic attenuation parameter increased almost linearly over development time in culture, with no significant differences between constructs in control and osteogenic media. Since the acoustic attenuation is typically an indicator of increased acoustic impedance and/or scatterer concentration, this increase may indicate cell proliferation and/or mineral deposition. These results of construct volume, speed of sound, and acoustic attenuation were used in further analysis of the constructs including estimation of spectral parameters and calcium deposits by the differentiated cell constructs in osteogenic media.

### SUSI analysis of Size of Cells or Scatterers in Constructs

**Figure 5:**
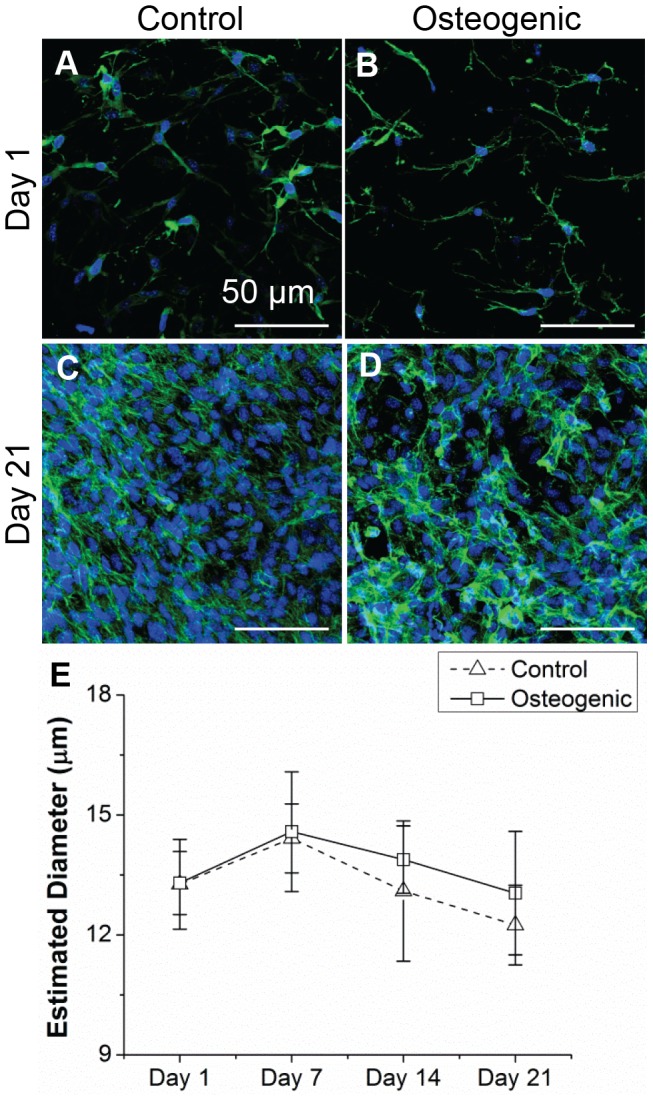
Developmental changes in sizes of MC3T3 cells seeded in collagen constructs. (A) –(D) Fluorescence staining of MC3T3 cells embedded in collagen constructs in control and osteogenic media on day 1 and day 21 (actin cytoskeleton is stained green, nuclei are stained blue). (E) Estimated diameter of cells from SUSI analysis over time in culture. Scale bar  = 50 µm. Best viewed in color.

Figure 5A–D shows the seeded MC3T3 cells in the collagen hydrogels with the F-actin filaments stained in green and cell nuclei stained in blue. Fluorescent imaging was performed on multiple slices of the hydrogel and was observed to be consistent across slices. The central region of the construct was chosen to represent the images in Figure 5A–D. From these images, diameters of the cell nuclei were estimated using a customized MATLAB script to be 6.0±1.0 µm (*n* = 4) on day 1 and 7.6±1.9 µm (*n* = 4) on day 21; these values are not statistically different. The effective size of the cells over the three week period was also estimated using the slope parameter obtained from SUSI analysis (Figure 5E). The average diameter for the cells, was approximately 14 µm with a range from about 12–15 µm, and remained essentially unchanged during the three week culture period for constructs in both control and osteogenic media. The significantly higher estimate value for scatterer diameter from SUSI compared to nucleus diameter measurement from DAPI stain suggest that the entire cell may have involved in the scattering of ultrasound, not merely cell nucleus.

### Acoustic Concentration and Calcium Deposition in Constructs

**Figure 6:**
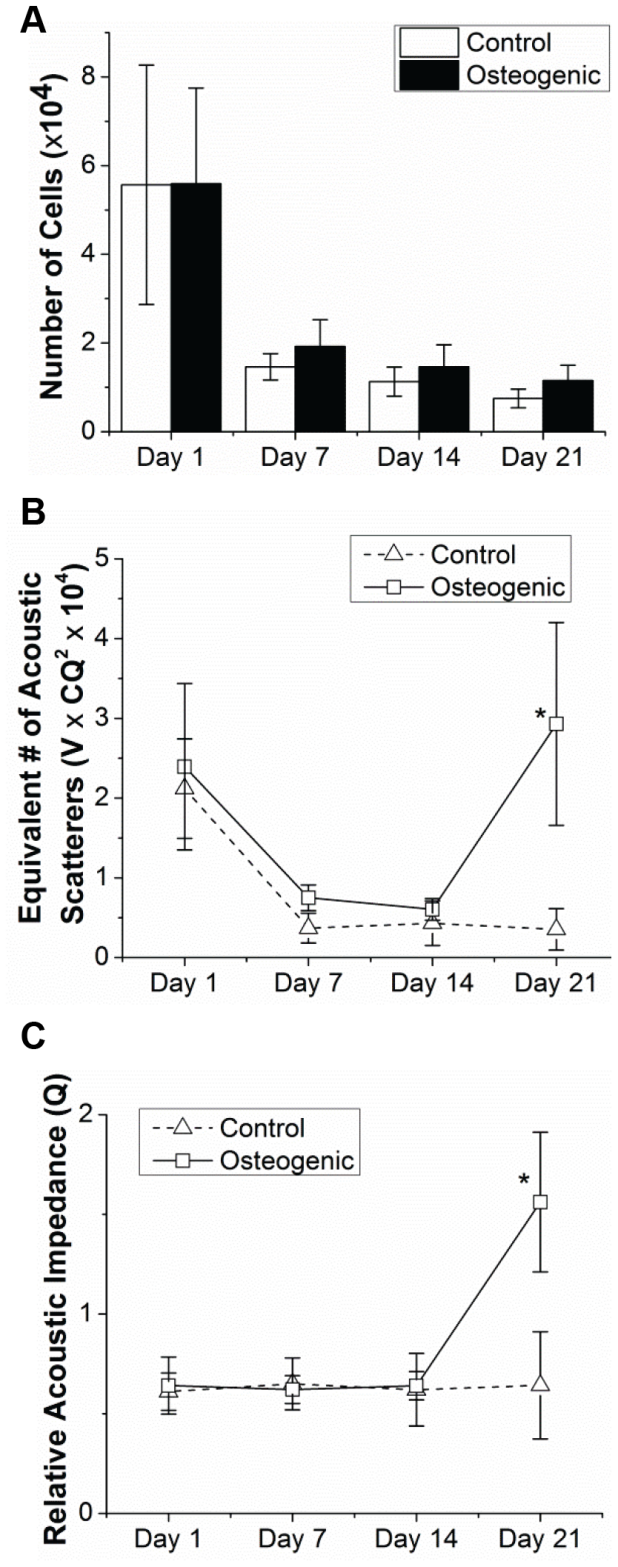
Quantified development of MC3T3 cells seeded in collagen constructs (A) Total number of cells as assessed by DNA quantification of MC3T3-seeded collagen constructs in control and osteogenic media over time in culture. (B) Equivalent number of acoustic scatterers as estimated from SUSI analysis. (C) Relative acoustic impedance estimated from (A) and (B).

The total amount of DNA in a construct was measured biochemically and converted to the total number of cells by determining the average amount of DNA per cell on a construct at day 0, when the number of cells was known (0.5×10^6^ cells/construct). As shown in Figure 6A, the number of cells decreased by about 60% from day 1 to day 7 in constructs in both the control and osteogenic groups, indicating probable cell death or migration out of the constructs. Thereafter the number of cells remained constant from days 7 to 21 with no significant differences between or within media groups. These cell numbers correlated well with the equivalent number of acoustic scatterers, which is the acoustic concentration (CQ^2^) estimated by SUSI analysis multiplied by the construct volume, with the exception of the day 21 measurement in the osteogenic group (Figure 6B). The acoustic concentration from SUSI depends on both the relative acoustic impedance of the scatterers (Q) and the actual number of scatterers (C). Thus assuming the actual number of scatterers or cells remained constant, the increased equivalent number of acoustic scatterers (or increased acoustic concentration) can be attributed to an increase of the relative acoustic impedance of the scatterers during the last days of incubation of the constructs (Figure 6C). The significantly increased acoustic impedance on day 21may therefore be indicative of changes due to the differentiation process in the constructs. Since acoustic impedance depends on the mass density and the speed of sound in scatterers, the increased acoustic impedance may reflect an increase in mass density due to calcium deposition associated with cell differentiation. As calcium is much denser than water, its presence is expected to significantly increase the relative acoustic impedance of the scatterers.

**Figure 7:**
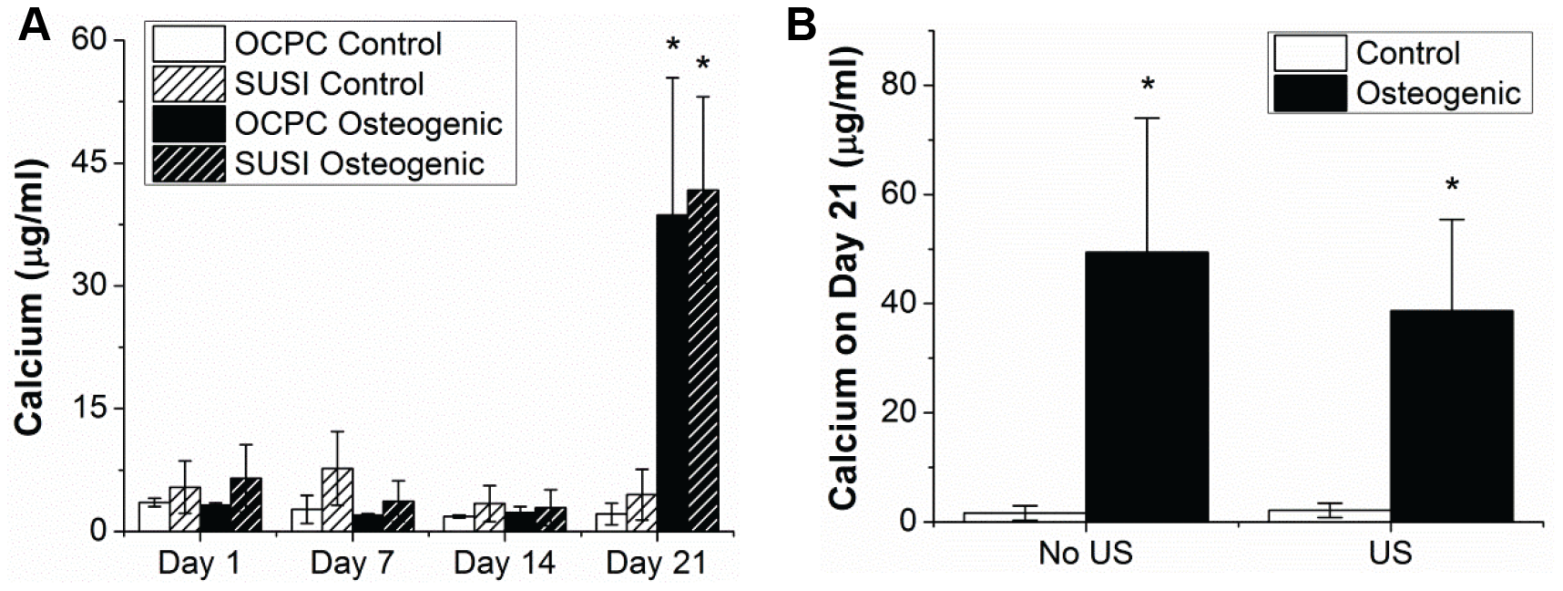
Amount of calcium mineral secreted by MC3T3-seeded collagen constructs in control and osteogenic media. (A) Calcium content as determined by OCPC assay and SUSI estimation. (B) Comparison of calcium content between constructs cultured in control and osteogenic media with and without exposure to ultrasound imaging.

As a quantitative marker to identify the extent of osteogenic differentiation of seeded MC3T3 cells [36], we estimated the calcium content using both standard biochemical assays (OCPC method) and SUSI. The estimated calcium concentration from SUSI on day 21 was 41.7±11.4 µg/ml (*n* = 9) and was comparable with the measured values of 38.7±16.7 µg/ml (*n* = 10) from the OCPC method. No statistically significant difference in calcium deposition at day 21 was detected in constructs subjected to ultrasound imaging and those without ultrasound imaging performed (Figure 7B).

### Spatiotemporal Evolution of Constructs by Parametric Ultrasound Imaging

**Figure 8:**
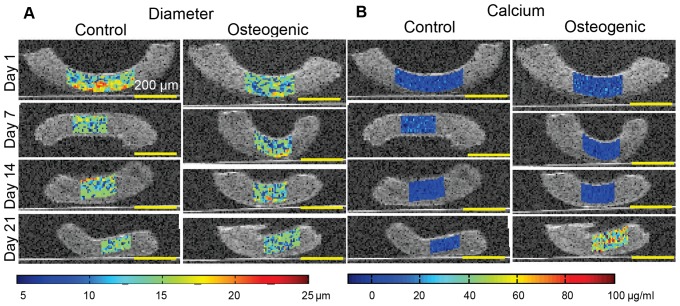
Overlaid B-mode (grayscale) and color maps of SUSI parameters. (A) Cell diameter, and (B) mass of calcium deposition of MC3T3 cells embedded in collagen constructs in control and osteogenic media. Best viewed in color.

The estimated microstructural properties (acoustic scatterer size and secreted calcium concentration) from SUSI analysis were used to generate parametric, color coded images overlaid on B-mode images, allowing visual assessment of the spatiotemporal evolution of constructs during development. As an example, Figure 8 shows the estimated microstructural parameters in a representative region of interest for constructs in control and osteogenic groups throughout the 21 day culture period. The representative region was chosen based on the bandwidth of the ultrasound focus (1.5–2.0 mm). Although, local variations in scatterer diameter estimation were observed, the average scatterer diameter of the construct did not vary significantly during the development process in either control or osteogenic medium (Figure 8A). The estimated calcium concentration was relatively constant at low values at all time-points to day 14, but exhibited a significantly higher value in the constructs in osteogentic medium on day 21. The increase in calcium content is indicative of osteogenic differentiation of MC3T3 cells in the constructs.

## Discussion

In this study, we demonstrated that high resolution ultrasound imaging provided non-destructive monitoring of MC3T3-seeded collagen constructs over 3 weeks. Physical parameters including the volume of each individual construct, speed of sound and acoustic attenuation in the constructs were obtained from simple analysis of the ultrasound RF signals. Notably, SUSI analysis provided estimation and assessment of key microstructural characteristics of the constructs, beyond what can be generated by conventional ultrasound images. These parameters included cell size, acoustic scatterer concentration, cell number, and mineral deposition. Since system-dependent factors are removed from SUSI analysis by calibration, the parameters provided are objective and instrument-independent. Therefore such data have broad utility and are particularly useful for inter-study comparisons. These features make ultrasound imaging and particularly SUSI a very attractive tool for biomaterials and tissue engineering research, and as a tool in quality assurance as engineered tissues approach the market. Below we discuss implications of our results and limitation of this study.

When embedded in 3D collagen hydrogels, many cell types including fibroblasts, smooth muscle cells, cardiomyocytes and osteoblasts, will remodel the collagen by exerting contractile forces that can align and compact the matrix [37]. These forces are significantly higher than the forces required for cell locomotion and it has been proposed that this force generation is targeted at matrix remodeling, rather than cell migration [38], [39]. This morphogenic phenomenon has been studied widely, and the mechanisms are still not fully understood. Assessment of tissue construct morphology in 3D in a non-invasive method is important to study these changes, and to quantitatively characterize the degree of remodeling. In this study, we showed that high resolution 3D ultrasound imaging could be used to noninvasively track morphological changes in tissue constructs longitudinally, revealing the significant compaction of unconstrained MC3T3-seeded constructs from day 1 to day 7.

We showed that ultrasound imaging provides non-destructive monitoring without affecting the structure or function of the constructs. Cell diameter as determined by SUSI was in the range of 12.5–15.5 µm, which matched the size determined by parallel fluorescent confocal imaging. SUSI analysis also revealed that the total number of acoustic scatterers in unconstrained MC3T3-seeded collagen constructs decreased by approximately 80% over the 21 day culture period in control medium. This result was in agreement with destructive biochemical DNA measurement performed in parallel. The decrease in cell number may have been a result of cell death or migration from the construct, possibly as a result of the decrease in construct volume that resulted from gel compaction. A similar pattern in cell number was observed in a previous study of unconstrained constructs seeded with undifferentiated mesenchymal stem cells (MSC) [40].

The acoustic concentration is defined as *CQ^2^*, where *C* is the number concentration of the scatterers and *Q* the acoustic impedance of the scatterers. The parameter *Q* depends on the physical and acoustic properties of the scatterer, particularly the mass density and speed of sound. Therefore assessment of the total number of acoustic scatterers (*CQ^2^* multiplied by construct volume) can provide information regarding changes in the construct microstructure. MC3T3 cells secrete mineral into the surrounding matrix as they undergo osteogenic differentiation [36]. They thereby modify the properties of the acoustic scatterers in the construct by increasing their mass density and thus the relative acoustic impedance. Therefore, relative acoustic impedance can be used as an indicator of changes in cellular state during osteogenic differentiation. In the current study, we qualitatively characterized the differentiation process by monitoring changes in the relative acoustic impedance, and also generated quantitative values of the mass of calcium deposited based on the relative acoustic impedance values. These data matched well with parallel measurement from conventional destructive biochemical tests for calcium content. We therefore have demonstrated that ultrasound imaging can be used to estimate the mass of calcium mineral in 3D collagen constructs as MC3T3 cells differentiate in osteogenic medium.

The current study highlights the advantages of high frequency ultrasound imaging for monitoring of tissue construct development. The use of a high frequency in our study provided high spatial resolution, which allowed detailed characterization of the constructs *in vitro*. However, most *in vivo* and eventual clinical applications will require the use of lower frequency imaging (e.g. 10 MHz) to allow deeper tissue penetration, which will reduce the resolution of the images. Another limitation of our current method is that our estimation of the relative acoustic impedance in this study required knowledge of the number of cells, which may not be readily available non-destructively. Relative changes to the acoustic concentration, a composite parameter that is obtained directly from SUSI analysis, can be used to noninvasively assess relative changes in the constructs. However, at this stage we are not able to use this parameter to generative an absolute quantitative estimation of calcium deposition without knowledge of the cell number. We assumed that cells and the calcium they deposited were a single scatterer, however future work will examine the ability to resolve different cell types and matrix components using SUSI.

## Conclusions

High resolution spectral ultrasound imaging (SUSI) was shown to provide non-destructive 3D imaging of *in-vitro* osteogenic differentiation of MC3T3 pre-osteoblasts seeded within collagen hydrogels. SUSI analysis enabled accurate measurement of construct volume and estimation of cell size, acoustic cell concentration, cell acoustic impedance, and calcium deposition within the construct. The technique was used to compare constructs cultured in maintenance and osteogenic media formulations, and SUSI data were validated by independent biochemical methods. Overlay of spectral parameter data on B-mode images allowed clear visualization of the spatiotemporal changes in construct composition. This study demonstrates that spectral ultrasound imaging can be a useful noninvasive tool to quantitatively characterize the development of orthopedic engineered tissues over time.

## Supporting Information

Figure S1 **Experiment for estimation of relative acoustic impedance of MC3T3 cells on day 0 and validation of estimated cell concentration from SUSI analysis.** (A)–(D) Ultrasound B-mode (grayscale) images of MC3T3-seeded collagen constructs on Day 0 at 0.5, 1, 2 and 5×10^6^ cells/ml cell concentrations, respectively. (E) Cell diameter, and (F) cell concentration estimated from SUSI analysis and compared to seeded cell concentration at day 0.
(DOCX)

Table S1 Tabular values of estimated Polybead® polystyrene microsphere (Polysciences Inc.) bead size and concentration and comparison with their true values.
(DOCX)

Appendix S1 **Derivation of the total mass of calcium on day** ***i*** **from the total number of scatterers (cells) and relative acoustic impedance.**
(DOCX)
