# Supplementary figures and images for: Noninvasive Quantification of In Vitro Osteoblastic Differentiation in 3D Engineered Tissue Constructs Using Spectral Ultrasound Imaging
Source: PLoS One. 2014 Jan 22;9(1):e85749. doi: 10.1371/journal.pone.0085749 (PMC3899074; doi:10.1371/journal.pone.0085749)

**
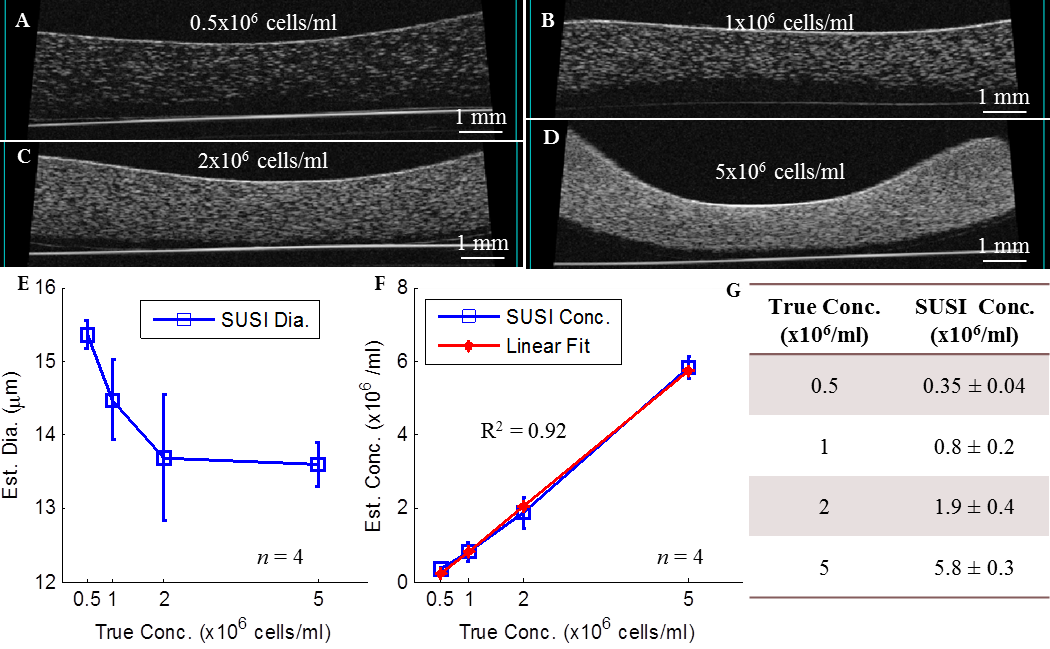
**

Supplement: Figure S1 — Experiment for estimation of relative acoustic impedance of MC3T3 cells on day 0 and validation of estimated cell concentration from SUSI analysis. (A)–(D) Ultrasound B-mode (grayscale) images of MC3T3-seeded collagen constructs on Day 0 at 0.5, 1, 2 and 5×10^6^ cells/ml cell concentrations, respectively. (E) Cell diameter, and (F) cell concentration estimated from SUSI analysis and compared to seeded cell concentration at day 0. (DOCX) [file pone.0085749.s001.docx]
