# Supplementary material for: Noninvasive Quantification of In Vitro Osteoblastic Differentiation in 3D Engineered Tissue Constructs Using Spectral Ultrasound Imaging
Source: PLoS One. 2014 Jan 22;9(1):e85749. doi: 10.1371/journal.pone.0085749 (PMC3899074; doi:10.1371/journal.pone.0085749)
Supplement: Table S1 — Tabular values of estimated Polybead® polystyrene microsphere (Polysciences Inc.) bead size and concentration and comparison with their true values. (DOCX) [file pone.0085749.s002.docx]

| **Size (µm)** | | **Concentration (x10^4^ beads/mm^3^)** | | | | | | | |
| --- | --- | --- | --- | --- | --- | --- | --- | --- | --- |
|  |  | **6 µm** | | **10 µm** | | **16 µm** | | **25 µm** | |
| **True** | **Est** | **True** | **Est** | **True** | **Est** | **True** | **Est** | **True** | **Est** |
| 6 | 6.4 ± 0.2 | 7.1 | 6.2±0.2 | 1.4 | 1.1±0.2 | 0.6 | 0.4±0.1 | 0.22 | 0.13±0.04 |
| 10 | 10.1 ± 0.6 | 9.7 | 9.4±0.4 | 2.7 | 2.5±0.4 | 0.8 | 0.7±0.2 | 0.40 | 0.36±0.08 |
| 16 | 13.4± 1.1 | 15.4 | 16.6±0.8 | 3.9 | 4.4±0.8 | 1.5 | 1.6±0.3 | 0.52 | 0.56±0.11 |
| 25 | 24.7 ± 0.2 | 21.9 | 23.1±1.4 | 5.0 | 5.5±1.1 | 2.3 | 2.5±0.5 | 0.62 | 0.71±0.15 |
