# Supplementary material for: Noninvasive Quantification of In Vitro Osteoblastic Differentiation in 3D Engineered Tissue Constructs Using Spectral Ultrasound Imaging
Source: PLoS One. 2014 Jan 22;9(1):e85749. doi: 10.1371/journal.pone.0085749 (PMC3899074; doi:10.1371/journal.pone.0085749)
Supplement: Appendix S1 — Derivation of the total mass of calcium on day i from the total number of scatterers (cells) and relative acoustic impedance. (DOCX) [file pone.0085749.s003.docx]

**APPENDIX**

The relative acoustic impedance (*Q*) of the scatterers in the construct on day *i* is

$Q_{i} =\frac{\rho_{i}c_{i}-\rho c}{\rho c}$ (A1)

where *ρ_i_* and *ρ* are the densities of the scatterer and extracellular matrix (ECM), *c_i_* and c are the speed of sound in the scatterer and ECM respectively. Rewriting the expression for density of the net scatterer,

$\rho_{i}=\frac{\rho c}{c_{i}}\left(1+Q_{i}\right)$ (A2)

The density of the net scatterer can also be calculated from its total mass and volume. On any specific developmental time-point of the construct, the net scatterer is considered to be a cell along with any secreted calcium mineral. Thus, the total mass of the net scatterer on day *i* is the sum of the masses of a cell on day 0 and secreted mineral on day *i.*

$\rho_{i}=\frac{V_{0}\rho_{0}+m_{i}^{cal}}{V_{i}}$ (A3)

After rearranging the expression for mass of the mineral secreted by each cell, the total mass of calcium on day *i* can be obtained.

$M_{i}^{cal}=N_{i}m_{i}^{cal}=N_{i}\left(V_{i}\rho_{i}-V_{0}\rho_{0}\right)$ (A4)

$M_{i}^{cal} =N_{i}\rho c\left(\frac{V_{i}}{c_{i}}\left(1+Q_{i}\right)-\frac{V_{0}}{c_{0}}\left(1+Q_{0}\right)\right)$ (A5)

where *N_i_* is the total number of cells on day *i* and *V_i_* is the volume of the net scatterer on day *i* which is approximately $\frac{4}{3}\pi a_{i}^{3}$.
